# Phenotypic Expression of Known and Novel Hemoglobin A2-Variants, Hemoglobin A2-Mae Phrik [Delta 52(D3) Asp > Gly, HBD:c.158A > G], Associated with Hemoglobin E [Beta 26(B8) Glu > Lys, HBB:c.79G > A] in Thailand

**DOI:** 10.3390/genes13060959

**Published:** 2022-05-27

**Authors:** Amphai Phasit, Sitthichai Panyasai, Monthon Mayoon, Niphawan Jettawan, Surada Satthakarn

**Affiliations:** 1 Department of Medical Technology, Lampang Hospital, Lampang 52000, Thailand; ampai.1616@gmail.com (A.P.); mont4477@gmail.com (M.M.); niphawanje@yahoo.com (N.J.); 2 Unit of Excellence in Integrative Molecular Biomedicine, Department of Medical Technology, School of Allied Health Sciences, University of Phayao, Phayao 56000, Thailand; satthakarn.su@up.ac.th

**Keywords:** Hb A_2_-Mae Phrik, δ-globin variant, Hb E, deletional α^+^-thalassemia, β-globin haplotype

## Abstract

The interactions of δ-globin variants with α- and β-thalassemia or other hemoglobinopathies cause complex thalassemic syndromes and potential diagnostic problems. Understanding the molecular basis and phenotypic expression is crucial. Four unrelated Thai subjects with second hemoglobin (Hb) A_2_ fractions were studied. A standard automated cell counter was used to acquire initial hematological data. Hb analysis was carried out by capillary electrophoresis (CE) and high-performance liquid chromatography (HPLC) assays. Globin gene mutations and haplotype were identified by appropriate DNA analysis. An allele-specific polymerase chain reaction method was developed to provide a simple molecular diagnostic test. Hb analysis revealed a Hb A_2_ variant in all cases. DNA analysis of the δ-globin gene identified the Hb A_2_-Melbourne [δ43(CD2)Glu > Lys] variant in combination with Hb E in three cases. Analysis of the remaining case identified a novel δ-Hb variant, namely Hb A_2_-Mae Phrik [δ52(D3)GAT > GGT; Asp > Gly], found in association with Hb E and α^+^-thalassemia, indicative of the as yet undescribed combination of triple heterozygosity of globin gene defects. An allele-specific PCR-based assay was successfully developed to identify this variant. The β-haplotype of the Hb A_2_ Mae-Phrik allele was strongly associated with haplotype [+ − − − − ± +]. This study advanced our understanding of the phenotypic expression of known and novel δ-Hb variants coinherited with other globin gene defects, routinely causing problems with diagnosis. Therefore, knowledge and recognition of this Hb variant and molecular assessments are crucial to improving diagnosis.

## 1. Introduction

Hemoglobin A_2_ (Hb A_2_) is one of the forms of hemoglobin (Hb) that is produced in normal adult red blood cells. The Hb A_2_ tetramer is composed of two α chains and two δ chains (α_2_δ_2_) and is naturally expressed at a low level of 2.5–3.5% of the total hemoglobin in healthy adult life [1]. An increase in Hb A_2_ levels, usually to above 4%, is a potential marker for diagnosing the β-thalassemia (β-thal) trait [2]. δ-Hb variants (Hb A_2_ variants) result from nucleotide substitutions or deletions in either coding or non-coding regions of the δ-globin gene, structural alteration, or instability and may consequently affect the δ-globin chain synthesis. Since the δ-globin chain is synthesized at a reduced rate, the number of variants within it that are detected by several methods is far lower than that of the α- or β-globin genes.

Recently, about 97 structural δ-globin gene variants have been described worldwide, varying in different regions and different ethnic populations (http://globin.cse.psu.edu (accessed on 17 April 2022)) [3]. Nearly all are silent and have an innocuous clinical presentation in heterozygous and even homozygous cases. Although most are non-pathological variants, coinheritance with β- or α-thalassemia or other variants results in diverse clinical syndromes. This may lead to diagnostic problems, as the amount of Hb A_2_ observed is often reduced to a value within the range usually observed in normal individuals [4,5,6]. These δ-Hb variants are especially prevalent in the Mediterranean region and are occasionally seen in other regions such as Southeast Asia [7,8,9]. In Thailand, where thalassemias and Hb variants are prevalent, about 20–30% of the population carry the α-thalassemia gene, reaching 30–50% in the northern part of the country, whereas about 3–9% are β-thalassemia carriers. The average frequency of Hb E (HBB:c.79G > A) is 20–30%, reaching 50–60% at the junction of Thailand, Laos, and Cambodia. The prevalence of the Hb constant spring (CS) (HBA2:c.427T > C) or Hb Paksé (HBA2:c.429A > T) variants is 1–8% [10].

The actual epidemiology of δ-globin gene defects in Thailand has not been described. However, many δ-Hb variants have been identified, including Hb A_2_-Melbourne [δ43(CD2)Glu > Lys; HBD: c.130G > A] [11], Hb A_2_-Lampang [δ47(CD6)Asp > Asn; HBD: c.142G > A] [12], Hb A_2_-Kiriwong [δ77(EF1)His > Arg; HBD: c.233A > G] [13], Hb A_2_’ [δ16(A13)Gly > Arg; HBD: c.49G > C] [14], Hb A_2_-Walsgrave [δ52(D3) Asp > His; HBD:c.157G > C] [15], Hb A_2_-Indonesia [δ69(E13) Gly > Arg; HBD:c.208G > C], and Hb A_2_-Troodos [δ116(G18) Arg > Cys; HBD:c.349C > T] [16]. Moreover, these variants have also been described in association with Hb E and α- or β-thalassemias [5,10,14,17]. This indicates that δ-globin gene defects are fairly common in the region, and recognition of the presence of this variant is particularly important.

Despite the fact that these variants are readily detected by high-performance liquid chromatography (HPLC) and capillary electrophoresis (CE) by the observation of the extra Hb A_2_ fraction split from normal Hb A_2_, these fractions show similar mobility when assessed by both HPLC and CE assays. Thus, accurate identification and differential diagnosis should be performed by DNA analysis. Moreover, the clinical consequences and significance of many δ-Hb variants when coinherited with α- or β-thalassemia or other hemoglobinopathies remain unrecognized. To fully understand the variants’ hematological manifestations and molecular basis, we determined the mutations responsible for the novel δ-Hb variant, namely Hb A_2_-Mae Phrik, detected based on different Hb A_2_ fractions on electropherograms or chromatograms, and evaluated their influence on the hematological profile alongside the development of a simple molecular diagnostic tool.

## 2. Materials and Methods

### 2.1. Subjects and Hematological Studies

After informed consent was obtained, peripheral blood anticoagulated with ethylenediaminetetraacetic acid was collected from four unrelated Thai individuals (S1–S4) and the parents of subject 1 (S1). The subjects were initially diagnosed as Hb E heterozygotes and were also suspected of having δ-globin structural variants due to the presence of a second Hb A_2_ fraction, referring to the School of Allied Health Sciences, University of Phayao, for further Hb and DNA analyses. Hematological data were collected using an automated blood cell counter (Coulter UniCel DxH 800; Beckman-Coulter Co., Miami, FL, USA). Hemoglobin analysis was performed by capillary electrophoresis (Capillarys 2 Flex Piercing; Sebia, Lisses, France) and cation exchange HPLC on two dedicated devices (VARIANT II^TM^, β-Thalassaemia Short Program; Bio-Rad Laboratories, Hercules, CA, USA; and Premier resolution from Trinity Biotech, Bray, Ireland, USA). Iron deficiency status was determined by measuring the levels of serum iron (SI), serum ferritin, total iron-binding capacity (TIBC), and transferrin saturation (Tsat) using an automated immunoassay analyzer (ARCHITECT^®^ i2000SR, Abbott Laboratories, Abbott Park, IL, USA).

### 2.2. DNA Analysis

**Figure 1:**
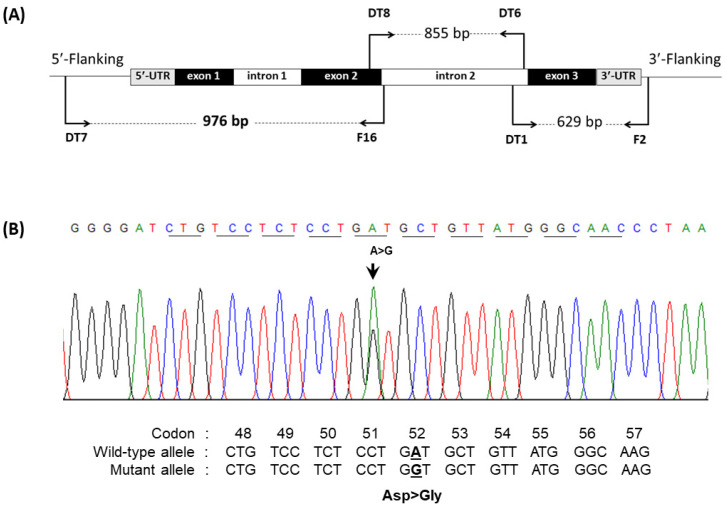
(**A**): Schematic drawing of the whole δ-globin gene, showing the 5′ untranslated, encoding and 3′ untranslated regions, and the locations and orientations of the specific primers that produced the overlapping fragments of the δ-globin gene. (**B**) Depiction of part of the DNA sequence of the δ-globin gene. The forward sequence demonstrates the substitution of a single nucleotide (A to G) at codon 52 of exon 2 (indicated by an arrow), resulting in a substitution of aspartate for glycine, responsible for Hb A_2_-Mae Phrik.

Genomic DNA was extracted from peripheral blood leukocytes using a genomic DNA isolation kit (BIO-HELIX Co., LTD., Keelung City, Taiwan). Identification of common α-thalassemia, including α-thalassemia 1 (SEA and THAI deletions), α-thalassemia 2 (−α^3.7^ and −α^4.2^), Hb constant spring (α^CS^), and Hb Paksé (α^Paksé^), mutations was performed routinely in our laboratory using the gap polymerase chain reaction (PCR) and allele-specific PCR (ASPCR) methods as previously described [18,19,20]. Hb A_2_-variants have previously been documented in Thai people, including the Hb A_2_-Melbourne (HBD:c.130G > A), Hb A_2_’ (HBD:c.49G > C), and Hb A_2_-Lampang (HBD:c.142G > A) variants, which were identified using multiplex ASPCR [12], alongside the Hb A_2_-Walsgrave (HBD:c.157G > C) variant, which was determined by ASPCR [15]. The β^E^ mutation at codon 26 (GAG to AAG) of the β-globin gene was investigated using an ASPCR method [21]. The amplification of the nucleotide sequence of the δ-globin gene using six specific primers generated three overlapping fragments (Figure 1A). The first amplified fragment (976 bp) was generated from position −408 of the Cap site to +568 using primers DT7 (5′- ATCTCTAGAGGCAAAGAAGA-3′) and F16 (5′-GAGCAGGTAGGTAAAAGAAC-3′) [11]. The second fragment (855 bp) was generated from position +408 to +1263 using primers DT8 (5′-TCACCTGGACAACCTCAAG-3′) and DT6 (5′-CCCATCAGCATAAATAAGTACAT-3′). The third fragment (629 bp) was generated from position +1170 to +1799 using primers DT1 (5′-AGACTACATGCTAGTTAAGT-3′) and F2 (5′-GTGTCACCCATTAATGCCTTGTAC-3′) [11]. Direct DNA sequencing of amplified δ-globin gene fragments was performed with an ABI PRISM^TM^ 3730 XL analyzer (Applied Biosystems, Foster City, CA, USA) after the samples were purified using the GF-1 AmbiClean Kit (Vivantis nucleic acid extraction kit, Malaysia) according to the manufacturer’s instructions. The variants have been named, following registration in HbVar [3], according to the classic nomenclature as specified by HGVS recommendations [22].

### 2.3. β-Globin Gene Haplotype Analysis

Globin gene haplotypes based on seven polymorphic restriction sites within the β-globin gene cluster, including HincII at 5′-ε, HindIII at ^G^γ, HindIII at ^A^γ, HincII at ψβ, HincII at 3′ψβ, AvaII at 5′β, and BamHI at 3′β, were determined using PCR and then restriction digestions, as described previously [23]. The haplotypes were documented as + or − according to the presence or absence of the respective restriction enzyme sites.

### 2.4. Development of An Allele-Specific Polymerase Chain Reaction Assay (ASPCR) for Identification of the Hb A_2_-Mae Phrik Variant

A novel δ-globin gene mutation (GAT > GGT) at codon 52 of exon 2 was identified in subject 1 (S1) and his mother, generating the Hb A_2_-Mae Phrik variant. This variant was confirmed using a newly developed ASPCR technique. The δ^52A > G^ allele-specific reverse primer SP35 (5′-AGGGTTGCCCATAACAGCAC-3′) was used with forward primer G58 (5′-AGGGCAAGTTAAGGGAATAG-3′) located 5′ upstream to produce a 500 bp fragment specific to the δ^52A > G^ allele. As an internal control for the PCR amplification, the 713 bp fragment was generated using primers G58 and F16. The PCR reaction mixture (50 μL) contained 50–200 ng of genomic DNA, 30 pmoles of primers G58 and F16, 7.5 pmoles of primer SP35, 200 μM of dNTPs, and 1.0 unit of *Taq* DNA polymerase (Vivantis Technologies, Selangor Darul Ehsan, Malaysia) in 10 mM Tris-HCl (pH 9.1) buffer, 50 mM KCl, and 0.1% TritonTM X100. The amplification reaction was carried out on a thermal cycler (Cyclerus personalis, Bio-Rad, USA). After an initial denaturation step of 94 °C for 3 min, the reaction was followed by 35 cycles of 94 °C for 1 min, 67 °C for 45 s, 72 °C for 1 min, and final extension at 72 °C for 10 min. The amplified product was analyzed by electrophoresis on a 1.5% agarose gel and visualized under UV light after ethidium bromide staining.

## 3. Results

**Figure 2:**
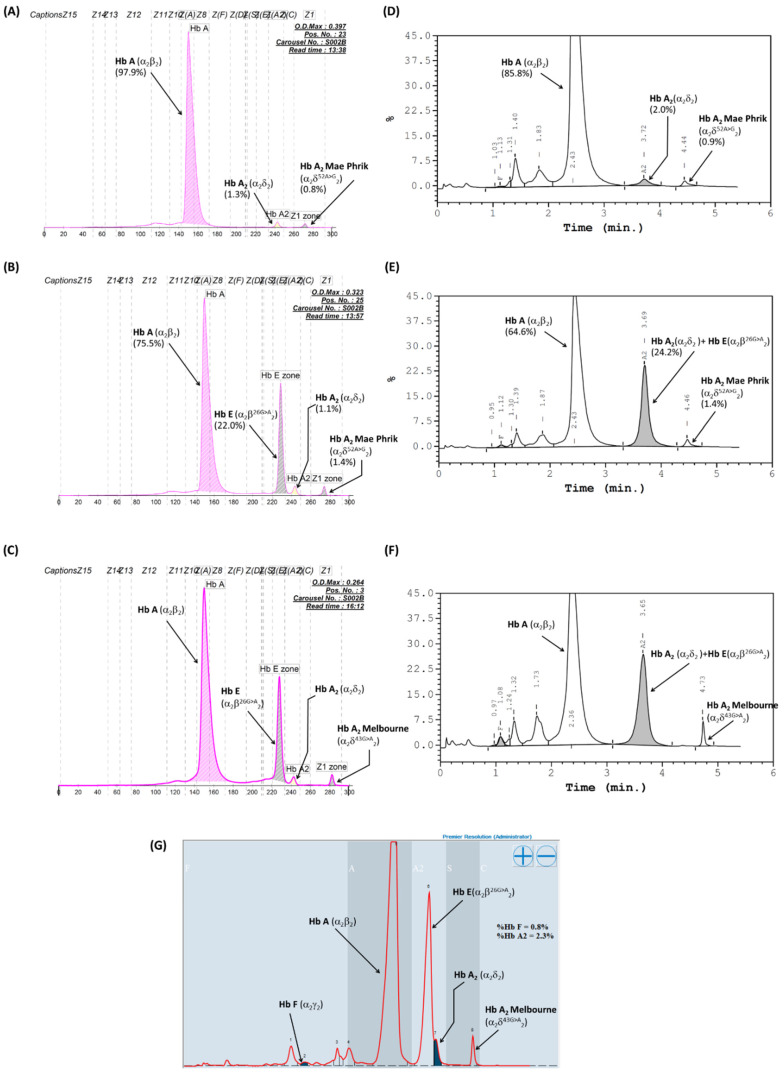
Representative Hb separation profiles of Hb A_2_-Mae Phrik using an automated Hb analyzer. (**A**,**D**): Electrophoretic and chromatographic mobility pattern of heterozygous Hb A_2_-Mae Phrik, observed in mother of subject 1. Hb A_2_-Mae Phrik migrates into the first position of the migration zone (**A**) and was eluted from the column after Hb A_2_ at a retention time of 4.44 min (**D**). (**B**,**E**): The Hb pattern of compound heterozygous Hb A_2_-Mae Phrik and Hb E observed in subject 1. Hb E and Hb A_2_ were completely separated, and a small peak of Hb A_2_-Mae Phrik also migrates to zone 1 on the electropherogram (**B**), while the HPLC chromatogram shows that Hb E and Hb A_2_ were eluted at the same retention time and Hb A_2_-Mae Phrik was completely separated from these, being eluted at a retention time of 4.46 min. (**C**,**F**,**G**): Electrophoretic (**C**) and chromatographic (**F**,**G**) mobility pattern of coinherited Hb A_2_-Melbourne with Hb E. The separated profiles of Hb A, Hb E, Hb A_2_, and A_2_-Melbourne are depicted.

The four unrelated subjects were initially diagnosed with heterozygous Hb E, coinherited with Hb A_2_ defects. Hematological characteristics, iron profiles, and globin genotypes of the four subjects (S1–S4) are summarized in Table 1. Hemoglobin analysis of S2, S3, and S4 using automated HPLC on a VARIANT II^TM^ system reveals a major peak of Hb A and Hb E and a small peak resembling the Hb A_2_-variant at a retention time of 4.73–4.75 min (Figure 2F), with the amounts ranging from 1.3 to 1.7%. Hb analysis using a capillary electrophoresis system showed similar patterns in all cases, in which the Hb A_2_-variant was clearly separated in zone 1 (Figure 2C), with the amounts ranging from 1.3 to 1.7%, while the Hb A_2_ levels were 1.0–1.4%, lower than those observed in heterozygous Hb E (3.8 ± 0.3%). Automated HPLC using the Premier Resolution system, which can separate Hb E and Hb A_2_, was recently installed in our laboratory during the study period. Hb analysis with this system was performed on the blood specimens of S2, S3, and S4. As shown in Figure 2G, Hb A, E, A_2_, and A_2_-variant were clearly separated on this system, allowing calculation of their amounts. The Hb A_2_-variant was eluted from the column at a retention time of 6.401–6.428 min, occupying the S zone of the chromatogram. The levels of Hb A_2_-variants were 1.4% in S2, 1.8% in S3, and 1.9% in S4. In all cases, DNA analysis by ASPCR revealed a G-A substitution at codon 43 of the δ-globin gene to be responsible for Hb A_2_-Melbourne, which has been previously documented in Southeast Asia [6,11,17]. Additionally, the analysis found that the subjects were positive for the β^E^-globin gene, while none of the common α-thalassemia genes mentioned above were observed. Consequently, the subjects were double heterozygotes at Hb A_2_-Melbourne and Hb E.

S1 was a 34-year-old male who attended an antenatal care clinic. Complete blood count and iron deficiency profiles revealed slightly decreasing mean corpuscular volume (MCV) and mean corpuscular hemoglobin (MCH) but no anemia or iron deficiency (Table 1). The Hb analysis using HPLC on the VARIANT II^TM^ system revealed a major peak of Hb A (64.6%) and Hb E (24.2%) and a small peak resembling Hb A_2_-variant (1.4%), with a retention time of 4.46 min on the HPLC chromatogram (Figure 2E). Additionally, analysis using capillary electrophoresis showed 75.5% Hb A, 22.2% Hb E, 1.1% Hb A_2_, and 1.4% probable HbA_2_-variant migrating toward the cathode lower than Hb A_2_, which was at the electrophoretic zone 1 (Figure 2B). The marked appearance of a small Hb peak on both the electropherogram and chromatogram in the subject prompted us to investigate structural abnormalities in the δ-globin chain. Analysis of the δ-globin gene performed using PCR and direct DNA sequencing revealed a novel single-nucleotide substitution in exon 2, HBD:c.158A > G (codon 52, GAT > GGT), shown in Figure 1B, resulting in a substitution of aspartate for glycine in the δ-globin chain. This mutation has not yet been described in the δ-globin gene [3], and we decided to name the new variant Hb A2-Mae Phrik after the region of origin of the subject. Further DNA analysis of the α- and β-globin genes revealed 3.7 kb deletional α^+^-thalassemia and also β^26G > A^ mutation, corresponding to Hb E; therefore, this subject was a triple heterozygote for Hb A_2_-Mae Phrik, Hb E, and deletional α^+^-thalassemia.

To determine whether the two variants were in cis or in trans, his parents were invited to participate in testing. Their hematological and iron deficiency profiles and globin genotypes are presented in Table 1. The father showed no anemia or alteration of the red blood cell indices, whereas mild anemia and slightly low MCV and MCH were observed in the mother. However, her iron profiles indicated normal iron storage. It was, therefore, less likely that she had iron deficiency but that the true reason for her anemia was not verified, and she may have had chronic disease anemia. Hb and DNA analysis revealed that the patient’s father carries a Hb E variant, accompanying α^+^-thalassemia. The mother’s Hb type according to HPLC was A (85.8%), A_2_ (2.0%), and A_2_-variant (0.9%) (Figure 2A), while techniques using CE demonstrated a similar Hb pattern (97.9% Hb A, 1.3% Hb A_2_, and 0.8% Hb A_2_-variant) (Figure 2D), constituting double heterozygosity for Hb E and Hb A_2_-variants.

δ-globin gene sequence analysis for the same condition investigated in S1 was performed in the mother. The analysis detected A and G at the same position in the δ-globin gene; she was therefore heterozygous for Hb A_2_-Mae Phrik. Further DNA analysis of the α-globin genes illustrated no abnormality and thus a possible genotype as αα/αα. The A-G mutation at codon 52 occupied the D helix of the δ-globin chain positioned on the molecular surface in the crystal structure of hemoglobin, which is not involved in contact with heme or between chains [24]. The β-globin gene haplotype analysis demonstrated that the presence of the Hb A_2_-Mae Phrik allele is associated with the β-globin gene haplotype [+ − − − − ± +] (Table 2).

**Figure 3:**
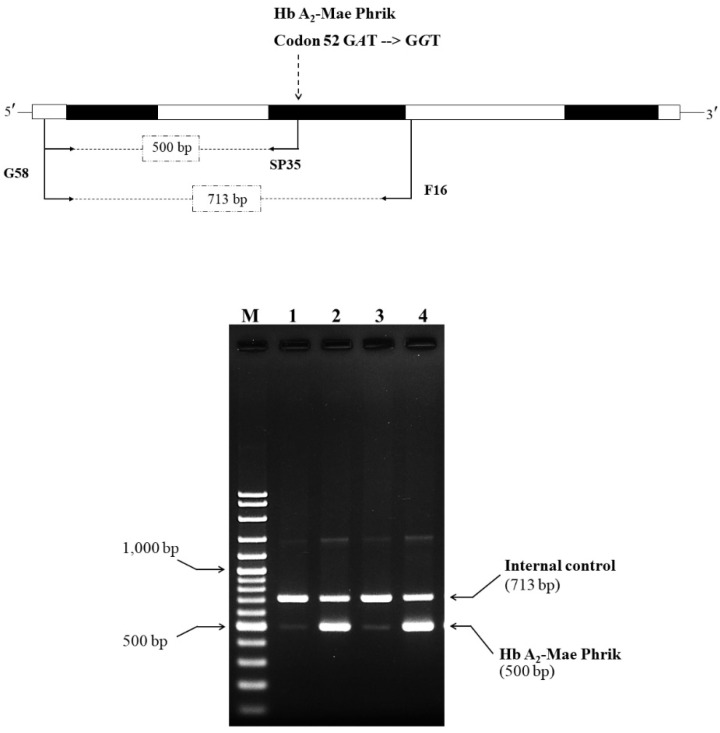
Identification of the Hb A_2_-Mae Phrik mutation by allele-specific PCR assay. The locations and orientations of primers G58 and F16 that produce the 713 bp specific fragment of the δ-globin gene. The Hb A_2_-Mae Phrik fragment is amplified by G58 and SP35, with a length of 500 bp. M represents the GeneRuler 100 bp Plus DNA marker. Lane 1: normal DNA; lanes 2 and 4: Hb A_2_-Mae Phrik; lane 3: normal individual.

We developed a new allele-specific PCR technique to confirm the presence of and provide a rapid diagnostic tool for Hb A_2_-Mae Phrik. As a result, a 500 bp amplified fragment specific to the δ^52A > G^ allele was observed in S1 and his mother, while a 713 bp fragment particular to the δ^A^ allele was observed in all cases (Figure 3). This result confirms that the patients have an A to G mutation of codon 52 of the δ-globin gene and that the newly developed ASPCR technique can be used successfully for the rapid diagnosis of the Hb A_2_-Mae Phrik variant.

## 4. Discussion

Many δ-Hb variants (Hb A_2_-variants) have been documented worldwide. Patients who are heterozygous or even homozygous for this variant usually have no clinically significant problems, since Hb A_2_ constitutes only a small proportion of the total Hb content. However, the interaction of δ-Hb variants with other variants or other thalassemias can lead to an erroneous interpretation of routine laboratory diagnostic test results [4,6,12,25]. This study reported known and novel Hb A_2_-variants coinherited with Hb E in Southeast Asia. Hb A_2_-Melbourne (HBD:c.130G > A), hitherto reported in patients from Thailand and Laos [11,17], was identified in the three Hb E heterozygote subjects (S2–S4). Mild microcytosis was observed in S2 and S4, while a remarkable reduction in MCV (69.2 fL) together with high red cell distribution width (RDW) was observed in S3, indicating masked α-thalassemia 1 gene. On the other hand, iron deficiency parameters and common α-thalassemia defects were not detected in all subjects (Table 1), demonstrating that the red cell alterations may affect Hb E. It is noteworthy that hematological parameters noted in these subjects were not likely to be more severe when compared with heterozygous Hb E, despite coinheritance of this variant [26]. The replacement of negatively charged glutamate with positively charged lysine results in an increased positive charge on the δ-globin chain surface. Thus, the net charge of the α_2_δ^43(G > A)^_2_ tetramer (Hb A_2_-Melbourne) was more positive compared to that of normal Hb A_2_, such that Hb A_2_-Melbourne was completely separated from Hb A_2_ by CE and HPLC methods, with Hb A_2_-Melbourne migrating more slowly than Hb A_2_ (Figure 2). In addition, the high resolution of the new HPLC device could distinguish this variant and allow calculation of its amount, showing a comparable result to the two methods mentioned above (Figure 2G). This facilitates easy detection of this variant during routine Hb analysis. Remarkedly, the amount of Hb A_2_-Melbourne observed in these three subjects, as measured using two assays (1.3–1.7%), was likely increased when compared to that observed using the same assay in definite cases of heterozygous Hb A_2_-Melbourne (0.9 ± 0.3%) [14]. This result is concordant with that previously noted in a pregnant Laotian woman [17]. The exact Hb A_2_ concentration could be calculated by integrating both of the split peaks of HbA_2_, generating a HbA_2_ concentration of 2.3% in S1, 2.9% in S2, and 3.1% in S3 for CE, which was the level usually observed in a group of normal individuals (2.5–3.5%) [1] but not in a group with heterozygous Hb E without concomitant iron deficiency or α-thalassemia; the Hb A_2_ concentration normally observed in such a group was 3.8–3.9% [26], the accepted range for diagnosing the β-thalassemia trait. Our findings demonstrate that the Hb A_2_ levels did not increase in production in compound heterozygous Hb E with the δ-Hb variant, indicating that the low total Hb A_2_ level reported in this condition might result from reduced synthesis or instability of the abnormal δ-globin chain [11]. We confirmed the low total Hb A_2_ value and that the second Hb A_2_ fraction is also visible in the presence of Hb E on Hb-CE analysis; the δ-globin gene mutation is the most likely diagnosis.

Hb A_2_-Mae Phrik (HBD:c.158A > G) is a novel δ-Hb variant characterized in S1, where it has been coinherited with Hb E (HBB:c.79G > A) and a deletional α^+^-thalassemia, with heterozygosity of this variant in the subject’s mother. Molecular analysis showed that S1 inherited the HBD:c.158A > G variant from his mother, while the HBB:c.79G > A variant was inherited from his father. This finding suggests that the β- and δ-globin gene mutations were carried in trans by the mother. The expression of hematological parameters of the two conditions was similar (Table 1) and did not significantly differ from those of the compound heterozygous Hb E and Hb A_2_-Melbourne. The replacement of glycine results in loss of one negative charge at the side chain of the δ-chain per molecule, leading to a marked change in the net charge of the tetramer of Hb A_2_-Mae Phrik. Consequently, the mobility of the α_2_δ^Mae Phrik^_2_ tetramer on CE and HPLC is slower than that of Hb A_2_ (α_2_δ^A^_2_), leading to it being completely separated from this variant and allowing measurement of the concentration of this variant (Figure 2). Electropherograms and chromatograms obtained from CE and HPLC, respectively, showed comparable results to HbA_2_-Mae Phrik. The level of HbA_2_-Mae Phrik, as measured using capillary electrophoresis, was 0.8% and 1.4% in a heterozygote and a compound heterozygote, respectively, while using HPLC revealed 0.9% and 1.4%, respectively. This demonstrated that HbA_2_-Mae Phrik levels were raised when combined with Hb E. In addition, we observed equal proportions of Hb A_2_ (1.3%) and Hb A_2_-Mae Phrik (0.8%), as measured using capillary electrophoresis, in the mother of S1, who was heterozygous for the variant. In addition, the variant combined with Hb E (S1) and Hb A_2_ and Hb A_2_-Mae Phrik was present in consistently equal proportions of 1.1% and 1.4%, respectively, indicating similar assembly rates of the two δ-globin chains to an α-globin chain. Of interest, the Hb A_2_(α_2_δ^A^_2_) level was observed to be 1.3% and 1.1%, although the latter was a value observed in S1 who had coinherited the variant with Hb E, which was likely to lead to a lower value when compared to the range normally observed in heterozygous Hb E without an α- or β-thalassemia allele [19]. However, in order to calculate the actual Hb A_2_ value in these cases, the combined percentage of Hb A_2_ and the Hb A_2_-Mae Phrik variant must be assessed. Therefore, total Hb A_2_ as measured by CE was 2.5% (1.1 + 1.4) in S1 and 2.1% (1.3 + 0.8) in his mother, who was heterozygous for this variant. This showed a consistently lower value than those usually observed for heterozygous Hb E (3.8 ± 0.3%) [19]. This result demonstrates a distinct decrease in total Hb A_2_ when combined with other Hb variants, which might result from the reduced synthesis of the abnormal δ-globin chain. Nevertheless, an aspartate amino acid at codon 52 of the δ-globin chain is in the second of the D helices of the Hb molecule and is not involved in contact with heme or between chains. Therefore, the replacement of glycine at this position could compromise the instability of the Hb molecule.

The characteristic HPLC retention times seen in Hb A_2_-Mae Phrik ranged from 4.44 to 4.46 min (Figure 2D,E), compared to the 4.73–4.75 min observed for HbA_2_-Melbourne. It seems that Hb A_2_-Mae Phrik eluted from the cation exchange column markedly faster than HbA_2_-Melbourne. This may be explained by the fact that glutamate was substituted for lysine in HbA_2_-Melbourne, providing increased affinity with the column than when aspartate was replaced with glycine in Hb A_2_-Mae Phrik. Therefore, we suggest that the characteristic HPLC retention times obtained for Hb A_2_-Melbourne and the novel variant Hb A_2_-Mae Phrik could be used to provide a presumptive identification.

Recently, another Hb variant caused by a mutation at the same position was identified, the non-pathological Hb A_2_-Walsgrave (δ52(D3) Asp > His; HBD:c.157G > C). This variant was found in the United Kingdom in a person who had emigrated from India [27] and had also been characterized in a pregnant Thai woman [15]. Moreover, five other δ-Hb variants have been documented in Thailand [12,13,14,16]. These variants presented on an electropherogram in zone 1, similarly to Hb A_2_-Mae Phrik, potentially causing issues in the routine diagnosis of this variant. Thus, a differential diagnosis of these conditions requires DNA analysis in order to provide an accurate diagnosis and appropriate genetic counseling. The newly developed allele-specific PCR method can readily detect the Hb A_2_-Mae Phrik mutation in δ-globin genes. Moreover, the second small Hb peaks appear in the electropherogram, not only indicating Hb A_2_-variants but also possibly unstable or degraded Hb. Identifying the Hb A_2_-variant is crucial for diagnosis and the exclusion of β-thalassemia or α-thalassemia, especially in areas where thalassemias are prevalent.

Haplotype information permits a better understanding of the origin and evolutionary relationships between populations worldwide. β-haplotypic analysis demonstrated that the Hb A_2_-Mae Phrik allele was greatly associated with haplotype [+ − − − − ± +] (Table 2). These findings indicate that the β-haplotype is very likely similar to that observed in the ancestral Thai 5’ haplotype [23]. This may imply that the herein-described mutation in the δ-globin gene has the same evolutionary origin. Moreover, the β-haplotype associated with Hb A_2_-Mae Phrik is likely that observed in Hb A_2_-Melbourne [11,14]; the Hb A_2_-Mae Phrik allele certainly has the same evolutionary origin.

## 5. Conclusions

This is the first report of the novel Hb A_2_-Mae Phrik variant, with two different genotypes in Thai people. This variant is clinically silent, and coinheritance of α-thalassemia does not change its phenotype. Hb A_2_-Melbourne combined with Hb E is also described. Interaction of Hb E with the two δ-globin variants leads to a decrease in total Hb A_2_ level and therefore could lead to misdiagnosis when inherited together with a β-thalassemia allele. A second small Hb fraction from these or other δ-globin mutations seen in the same zone of the electropherogram might lead to problematic interpretation during routine Hb analysis. The HPLC retention times of the δ chain variants are characteristic and useful in order to initially distinguish these variants. A newly developed ASPCR assay is able to readily detect and distinguish this variant from others, facilitating accurate diagnosis, appropriate treatment, and genetic counseling.

## Figures and Tables

**Table 1:** Hematological data and globin genotypes of the subjects carrying Hb A_2_-variants and the parents of the subject carrying Hb A_2_-Mae Phrik.

Parameters	Family 1					
	Mother	Father	S1	S2	S3	S4
Sex/Age (years)	67	68	Male/34	Male/57	Female/54	Female/36
RBC count (×10^12^/L)	4.45	5.29	5.67	4.38	5.46	4.98
Hb (g/dL)	10.8	13.8	14.5	11.5	12.4	12.5
Hct (L/L)	33.9	43.4	43.3	35.0	37.8	38
MCV (fL)	76.1	82.0	76.3	79.9	69.2	76.4
MCH (pg)	24.3	26.1	25.6	26.3	22.8	25
MCHC (g/dL)	31.9	31.8	33.5	32.9	32.9	32.8
RDW-CV (%)	12.3	12.7	11.9	12.1	27.8	14.7
CE-Hb Profile ^a^	A_2_A with A_2_-variant	EA	EA with A_2_-variant	EA with A_2_-variant	EA with A_2_-variant	EA with A_2_-variant
Hb A (%) ^a^	97.9	73.5	75.5	69.5	75.6	72.3
Hb A_2_ (%) ^a^	1.3	3.4	1.1	1.0	1.3	1.4
Hb E (%) ^a^	0	23.1	22.0	24.6	21.5	24.6
Hb F (%) ^a^	0	0	0	0	0	0
Hb A_2_-variant (%) ^a^	0.8	0	1.4	1.3	1.6	1.7
HPLC-Hb Profile ^b^	A_2_A with A_2_-variant	EA	EA with A_2_-variant	EA with A_2_-variant	EA with A_2_-variant	EA with A_2_-variant
Hb A (%) ^b^	85.8	62.6	64.6	58.1	62.3	61.2
Hb A_2_ + E (%) ^b^	2.0	26.8	24.2	22.2	26.2	26.9
Hb F (%) ^b^	0.2	0.6	0.6	1.8	0.9	1.3
Hb A_2_-variant (%) ^b^	0.9	0	1.4	1.3	1.4	1.7
Iron profile						
Ferritin (μg/L)	120.5	33.9	74.2	893.1	34.2	82.7
Serum iron (μg/dL)	65.3	48.4	130.8	159.1	54.1	82.3
TIBC (μg/dL)	262.9	320.8	349.4	227.9	333.0	363.7
%Tsat (%)	24.8	15.1	37.4	67.8	16.3	22.6
δ-globin genotype	δ^52A > G^/δ^A^	δ^A^/δ^A^	δ^52A > G^/δ^A^	δ^43G > A^/δ^A^	δ^43G > A^/δ^A^	δ^43G > A^/δ^A^
α-globin genotype	αα/αα	−α^3.7^/αα	−α^3.7^/αα	αα/αα	αα/αα	αα/αα
β-globin genotype	β^A^/β^A^	β^E^/β^A^	β^E^/β^A^	β^E^/β^A^	β^E^/β^A^	β^E^/β^A^

**Table 2:** β-Globin gene haplotypes associated with Hb A_2_-Mae Phrik in a Thai family. + and − represent the presence and absence of the restriction enzyme sites, respectively.

		β-Globin Haplotype (5′ > 3′)								
Subjects	β-Genotype	*Hinc*II5’ ε	*Hind*III^G^γ	*Hind*III^A^γ	*Hinc*II5’ ψβ	*Hinc*II3’ ψβ	*Ava*IIβ	*Bam*HI3’ β	Hb A_2_-Mae Phrik Linked Haplotype	
Proband	δ^52A > G^/δ^A^	[+/−]	[+/−]	[−/−]	[+/−]	[+/−]	[+/−]	[+/+]	δ^52A > G^ δ^A^	[+ − − − − ± +] [− + − + + ± +]
Mother	δ^52A > G^/δ^A^	[+/+]	[−/−]	[−/−]	[−/−]	[−/−]	[+/−]	[+/−]	δ^52A > G^ δ^A^	[+ − − − − ± +] [+ − − − − ± +]
Father	δ^A^/δ^A^	[+/−]	[+/−]	[−/−]	[+/−]	[+/−]	[+/−]	[+/−]	δ^A^ δ^A^	[− + −+ + ± +] [+ − − − − ± −]

## Data Availability

Not applicable.
